# Modulation of Inflammation in McCoy Cells by Zinc Nanoparticles Conjugated With β-Chitosan

**DOI:** 10.7759/cureus.68653

**Published:** 2024-09-04

**Authors:** Mampi Payra, Karthik Ganesh Mohanraj, Taniya Mary Martin, Meenakshi Sundaram K

**Affiliations:** 1 Zebrafish Facility, Department of Anatomy, Saveetha Dental College and Hospitals, Saveetha Institute of Medical and Technical Sciences (SIMATS) Saveetha University, Chennai, IND

**Keywords:** cytokines, green synthesis, cytotoxicity, mccoy cells, anti-inflammatory, β-chitosan nanoparticles

## Abstract

Introduction: This study investigated biosynthetically derived β-chitosan-derived zinc nanoparticles (β-Ch-Zn NPs) for their potential anti-inflammatory properties on McCoy cells. β-Ch-Zn NPs were synthesized using a green chemistry approach, and their characterization confirmed successful synthesis, appropriate size, and morphology. The study aimed to evaluate the cytotoxicity of β-Ch-Zn NPs and their effects on inflammatory responses in McCoy cells stimulated with lipopolysaccharide (LPS).

Methods: β-Ch-Zn NPs were synthesized and characterized using Fourier-transform infrared spectroscopy (FTIR), ultraviolet-visible spectroscopy (UV-Vis) spectroscopy, and X-ray diffraction (XRD) to confirm their structural and morphological properties. The cytotoxicity of β-Ch-Zn NPs was assessed using the 3-(4,5-dimethylthiazol-2-yl)-2,5-diphenyl-2H-tetrazolium bromide (MTT) assay at various concentrations to determine safe doses for subsequent experiments. To induce inflammation, McCoy cells were pretreated with β-Ch-Zn NPs at different concentrations before LPS stimulations. Gene expression analysis using quantitative real-time polymerase chain reaction was performed to measure the messenger RNA (mRNA) levels of proinflammatory cytokine.

Results: FTIR, UV-Vis spectroscopy, and XRD confirmed the successful synthesis of β-Ch-Zn NPs with the desired size and morphology. The MTT assay demonstrated concentration-dependent cytotoxicity of β-Ch-Zn NPs, indicating safety for cellular studies. Pretreatment with β-Ch-Zn NPs significantly downregulated the mRNA expression of proinflammatory cytokines. The nanoparticles effectively downregulate proinflammatory cytokines and promote anti-inflammatory pathways, as evidenced by the significant reduction in interleukin (IL)-2, IL-6, hypoxia-inducible factor, and nuclear factor kappa B expression in a dose-dependent manner.

Conclusions: This study demonstrated that biosynthetically derived β-Ch-Zn NPs exhibit potent anti-inflammatory effects in McCoy cells. These findings underscore the therapeutic potential of β-Ch-Zn NPs for treating inflammatory conditions and support further investigation into their in vivo efficacy and safety.

## Introduction

Inflammation is a fundamental biological response to harmful stimuli such as pathogens, damaged cells, or irritants. It is a protective mechanism involving immune cells, blood vessels, and molecular mediators to eliminate the initial cause of cell injury, clear out necrotic cells, and establish tissue repair. While acute inflammation is crucial for healing and recovery, chronic inflammation is associated with various diseases, including arthritis, cardiovascular diseases, diabetes, and cancer. Therefore, managing and controlling inflammation is crucial to treating these conditions. Recent advantages in nanotechnology have opened new avenues for developing innovative anti-inflammatory agents. In inflammatory diseases, nanoparticles can be descended to deliver unimplemented drugs directly to the affected tissues, thereby enhancing therapeutic efficacy and minimizing systemic side effects. Additionally, nanoparticles can be engineered to modulate human responses. They can be cotton swabs infused with liquids that interact with specific receptors on immune cells, thereby influencing cell activation and cytokine release. This targeted approach can help control excessive inflammation and promote the resolution of chronic inflammatory conditions [1]. β-chitosan, a derivative of chitin, gained attention in biomedical and pharmaceutical applications due to its biocompatibility, biodegradability, and nontoxicity. However, its poor soluble nature in water restricts bioavailability; in this context, nanoparticles, such as zinc, could help efficiently deliver those particles. Among these advancements, β-chitosan-derived zinc nanoparticles (β-Ch-Zn NPs) have emerged as a promising candidate due to their unique properties and potential therapeutic benefits. Chitosan, a biopolymer derived from chitin found in the exoskeletons of crustaceans, insects, and fungi, is renowned for its biocompatibility, biodegradability, and nontoxic nature. β-chitosan, a specific form of chitosan, exhibits enhanced solubility and biological activity compared to its α-counterpart, making it particularly suitable for drug delivery and therapeutic applications [2-4].

Incorporating metal nanoparticles, such as zinc, into β-chitosan matrices can further enhance its biological properties. Zinc is an essential trace element involved in numerous biological processes, including enzyme function, protein synthesis, and cell division. Zinc oxide nanoparticles (ZnONPs) have been extensively studied for their antimicrobial, anticancer, and anti-inflammatory properties. ZnONPs have shown promise in modulating inflammatory responses, partly due to their ability to influence the production and activity of cytokines, which are key mediators of inflammation. Moreover, zinc's antioxidant properties help scavenge reactive oxygen species (ROS), which are known to exacerbate inflammatory responses [5-7].

Despite the promising potential of β-Ch-Zn NPs, several gaps remain in our understanding. While ZnONPs have demonstrated anti-inflammatory effects, the specific mechanisms through which β-Ch-Zn NPs exert these effects, particularly in an in vitro model, are not yet fully elucidated. Specifically, there is a need to explore how β-Ch-Zn NPs modulate proinflammatory cytokines, influence key signaling pathways involved in inflammation, and impact oxidative stress markers [6]. The current study aims to address these research gaps by investigating the anti-inflammatory effects of biosynthetically derived β-Ch-Zn NPs on McCoy cells in vitro. McCoy cells, a fibroblast-like cell line derived from mouse connective tissue, are commonly used to evaluate the biological effects of various compounds, including nanoparticles. These cells are beneficial for studying inflammation because they produce a wide range of inflammatory mediators in response to stimuli such as lipopolysaccharides (LPS). McCoy cells are very helpful in inflammation research and serve as an excellent model for the signaling network between the cells and external stimuli such as different microorganisms, cancer cells, and chronic diseases [7,8]. The synthesis of β-Ch-Zn NPs involves a green chemistry approach using plant extracts as reducing and stabilizing agents. Characterization techniques such as Fourier-transform infrared spectroscopy (FTIR), ultraviolet-visible spectroscopy (UV-Vis), and X-ray diffraction (XRD) will be employed to confirm the successful synthesis of the nanoparticles and determine their shape, size, and crystalline structure. These steps ensure the consistency and reproducibility of the nanoparticles used in biological studies [9].

The cytotoxicity of β-Ch-Zn NPs will be evaluated using the 3-(4,5-dimethylthiazol-2-yl)-2,5-diphenyl-2H-tetrazolium bromide (MTT) assay, which measures cell viability based on the reduction of a tetrazolium compound to formazan by metabolically active cells. This assay provides an initial assessment of the biocompatibility of the nanoparticles and helps to determine the appropriate concentration range for subsequent anti-inflammatory studies, ensuring that the nanoparticles are not cytotoxic at the concentrations used for anti-inflammatory studies. Ensuring that the nanoparticles are not cytotoxic at the concentrations used for anti-inflammatory assays is essential, as cell death could confound the results. To assess the anti-inflammatory effects of β-Ch-Zn NPs, McCoy cells will be stimulated with LPS to induce an inflammatory response [10,11]. The expression levels of key proinflammatory cytokines, such as tumor necrosis factor (TNF-α), interleukin (IL)-2, and IL-6, will be measured using quantitative real-time polymerase chain reaction (qRT-PCR). These cytokines are central players in the inflammatory process, and their modulation by β-Ch-Zn NPs would indicate the nanoparticles' potential to reduce inflammation. The study will investigate the impact of β-Ch-Zn NPs on key signaling pathways involved in inflammation, such as the nuclear factor kappa B (NF-κB) and mitogen-activated protein kinase pathways. The NF-κB pathway is a key regulator of immune and inflammatory responses, controlling the expression of numerous proinflammatory genes [12-14]. In addition to cytokine production and signaling pathways, the study will examine the impact of β-Ch-Zn NPs on oxidative stress markers. Oxidative stress is closely linked to inflammation, as ROS can activate signaling pathways that produce proinflammatory cytokines. The antioxidant properties of zinc and chitosan may help in reducing ROS in McCoy cells treated with β-Ch-Zn NPs. This will be measured using fluorescence-based assays. The expression of antioxidant enzymes, such as superoxide dismutase and catalase, will be evaluated to understand the nanoparticles' effect on the cellular antioxidant defense system [15].

Overall, this study aims to provide comprehensive insights into the anti-inflammatory potential of biosynthetically derived β-Ch-Zn NPs on McCoy cells. By elucidating their effects on cytokine production, signaling pathways, and oxidative stress, the study seeks to establish β-Ch-Zn NPs as a promising therapeutic agent for managing inflammation. The findings from this study could pave the way for further research and development of β-Ch-Zn NPs for clinical applications in inflammatory diseases, contributing to the growing field of nanomedicine [12-16].

## Materials and methods

Green synthesis of β-Ch-Zn NPs

To synthesize β-Ch-Zn NPs, a zinc ion solution was prepared by dissolving 0.1 mM zinc nitrate in deionized water. A 0.1 mM β-chitosan solution was prepared separately. These solutions were mixed under constant stirring to ensure thorough homogenization. To initiate the formation of ZnONPs, a freshly prepared 0.1 M sodium hydroxide solution was added dropwise to the mixture while stirring vigorously. Stirring continued for 30 minutes to complete the nanoparticle formation and stabilize the β-Ch-Zn NPs. The resulting nanoparticle suspension was centrifuged at 10,000 rpm for 20 minutes to separate the nanoparticles from unreacted materials and impurities. The supernatant was discarded, and the nanoparticles were washed multiple times with deionized water to remove residual reactants, ensuring the purity and stability of the synthesized β-Ch-Zn NPs [17].

Characterization of β-Ch-Zn NPs

The biosynthetic approach for synthesizing β-Ch-Zn NPs was confirmed through various characterization techniques. FTIR spectroscopy revealed characteristic absorption bands corresponding to the functional groups of β-chitosan and zinc oxide. The presence of broad peaks around 3,420 cm^-1^ (O-H stretching), 2,920 cm^-1^ (C-H stretching), 1,650 cm^-1^ (C=O stretching), and 1,380 cm^-1^ (C-N stretching) indicated successful incorporation of zinc ions into the β-chitosan matrix. UV-Vis spectroscopy showed a distinct absorption peak around 350 nm, confirming the formation of ZnONPs. XRD analysis displayed diffraction peaks corresponding to the hexagonal wurtzite structure of ZnONPs [18].

Cell culture and treatment

For the study, McCoy cells (procured from the National Centre for Cell Science, India) were selected and cultured in appropriate media supplemented with fetal bovine serum and antibiotics under standard conditions (37°C, 5% CO_2_). The McCoy cells were treated with LPS (10 μg/mL, 24 hours) to induce inflammation. Subsequently, the cells were treated with β-Ch-Zn NPs at varying concentrations to determine the optimal dose through a dose-response curve. Experimental groups were established, including a control group, an LPS-induced inflammation group, and groups treated with β-Ch-Zn NPs at different concentrations [19].

Cell viability assay: MTT assay

The MTT assay evaluated the cytotoxic effects of β-Ch-Zn NPs on McCoy cells. The results demonstrated that β-Ch-Zn NPs exhibited efficacy on McCoy cells. For analyzing biocompatibility, different concentrations (10, 20, 30, 40, 50, 60, 70, 80, 90, and 100 μg/mL) of conjugated nanoparticles were assessed. The cell viability was recorded. Doxorubicin was used as a positive control with the above-mentioned concentrations. From the biocompatibility testing, two concentrations were calculated based on the half-maximal inhibitory concentration value of the conjugated nanoparticles (33 and 66 μg/mL) for the gene expression analysis. The statistical significance was analyzed using a two-way analysis of variance and Bonferroni post hoc test [4,13].

Gene expression analysis

The expression levels of IL-2, IL-6, hypoxia-inducible factor (HIF), and NF-κB were measured by qRT-PCR. McCoy cells were treated with β-Ch-Zn NPs (33 and 66 µg/mL) for 24 hours. Total RNA was extracted using the RNeasy Mini Kit (Qiagen, Hilden, Germany), and complementary DNA (cDNA) was synthesized using the iScript cDNA Synthesis Kit (Bio-Rad, Gurugram, India). qRT-PCR was performed using the SYBR Green PCR Master Mix (Applied Biosystems, Waltham, MA) on a StepOnePlus Real-Time PCR System (Applied Biosystems). The primers used for amplification were as follows: Bax: forward 5'-TCCACCAAGAAGCTGAGCGAG-3', reverse 5'-GTCCAGCCCATGATGGTTCTG-3'; BCl-2: forward 5'-GGGAGGATTGTGGCCTTCTTT-3', reverse 5'-TGAAGGAGCGCAACCGGA-3'; IL-2: forward 5'AGCAGCTGTTGATGGACCTACC-3', reverse 5'-AGTTGATGGACCTGGGAAAGG-3'; IL-6: forward 5'-CCAGGAGCCCAGCTATGAA-3', reverse 5'-CCAGGCAAGTCTCCTCATTGA-3'; TNF-α: forward 5'-GCCCAGACCCTCACACTCAG-3', reverse 5'-GCTACAGGCTTGTCACTCGG-3'. The relative expression levels of the target genes were normalized to glyceraldehyde-3-phosphate dehydrogenase and calculated using the 2^−ΔΔCt^ method [16].

Statistical analysis

All experiments were performed in triplicate, and the data were presented as mean ± standard deviation. Statistical analysis was conducted using the GraphPad Prism 8 software (GraphPad Software Inc., San Diego, CA). Differences between the groups were analyzed using the t-test and the Mann-Whitney U test. A p value of less than 0.05 was considered statistically significant. The statistical significance was represented as follows: * represents p < 0.05 (statistically significant), ** represents p < 0.01 (highly statistically significant), and *** represents p < 0.001 (extremely statistically significant) [1-7].

## Results

This study synthesized β-chitosan-derived ZnONPs (β-Ch-ZnO-NPs), and their anti-inflammatory effects were evaluated in McCoy cells. The analysis included the assessment of cell viability using MTT assay at different concentrations and expression of IL-2, IL-6, HIF, and NF-κB genes.

UV-Vis spectroscopy analysis

**Figure 1:**
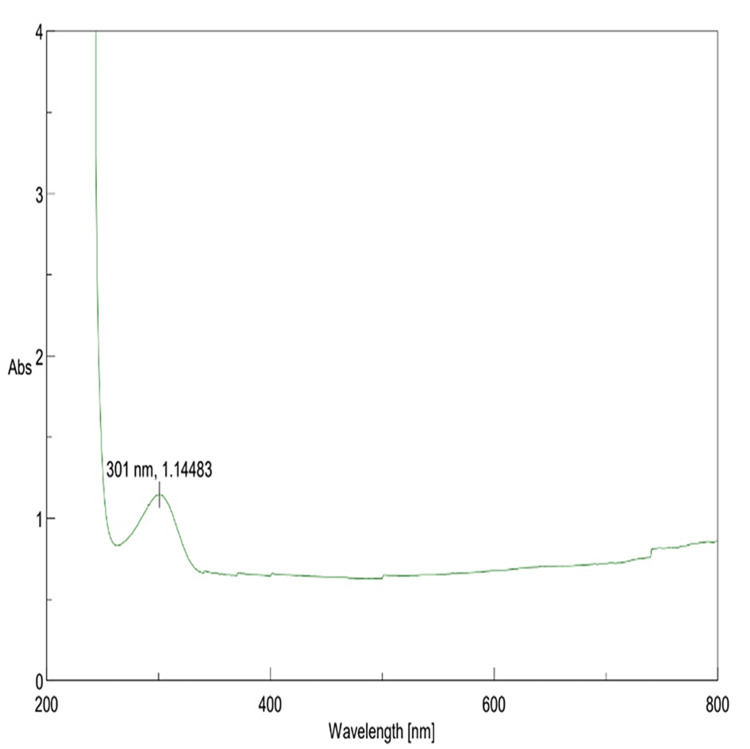
UV-Vis absorption spectra of β-Ch-ZnO-NPs UV-Vis: ultraviolet-visible spectroscopy; β-Ch-ZnO-NPs: β-chitosan-derived zinc oxide nanoparticles

β-Ch-ZnO-NPs were characterized using UV-Vis spectroscopy, revealing a distinct exciton band observed at 301 nm, closely resembling the characteristic exciton absorption of ZnONPs (350-373 nm). The rapid increase in absorbance upon excitation from the nanoparticles' ground state to their excited state further confirmed their optical properties. However, a subsequent decrease in radiation absorption suggested some nanoparticle agglomeration. These results highlight the successful synthesis of β-Ch-ZnO-NPs and their promising optical characteristics for various applications (Figure 1).

FTIR analysis

**Figure 2:**
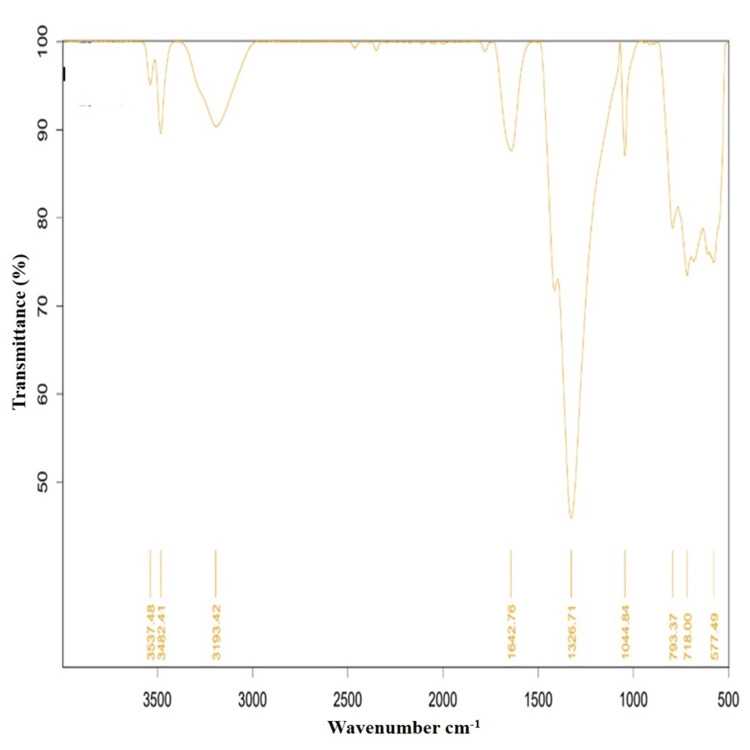
FTIR spectra of β-Ch-ZnO-NPs FTIR: Fourier-transform infrared spectroscopy; β-Ch-ZnO-NPs: β-chitosan-derived zinc oxide nanoparticles

FTIR analysis of biosynthesized β-Ch-ZnO-NPs was conducted to identify putative functional groups. As shown in Figure 2 of the infrared spectrum, a broad peak at 3,437 and 2,925 was related to the stretching vibration of hydroxyl compounds; 3,482 was attributed to the N-H stretch vibration of the amine functionality; 3,193 was related to the presence of =C-H stretch in the sample; and 1,642, 1,326, and 1,044 revealed the presence of -C=C aromatic, NO_2_ stretch, and C-OH stretch, respectively.

XRD analysis

**Figure 3:**
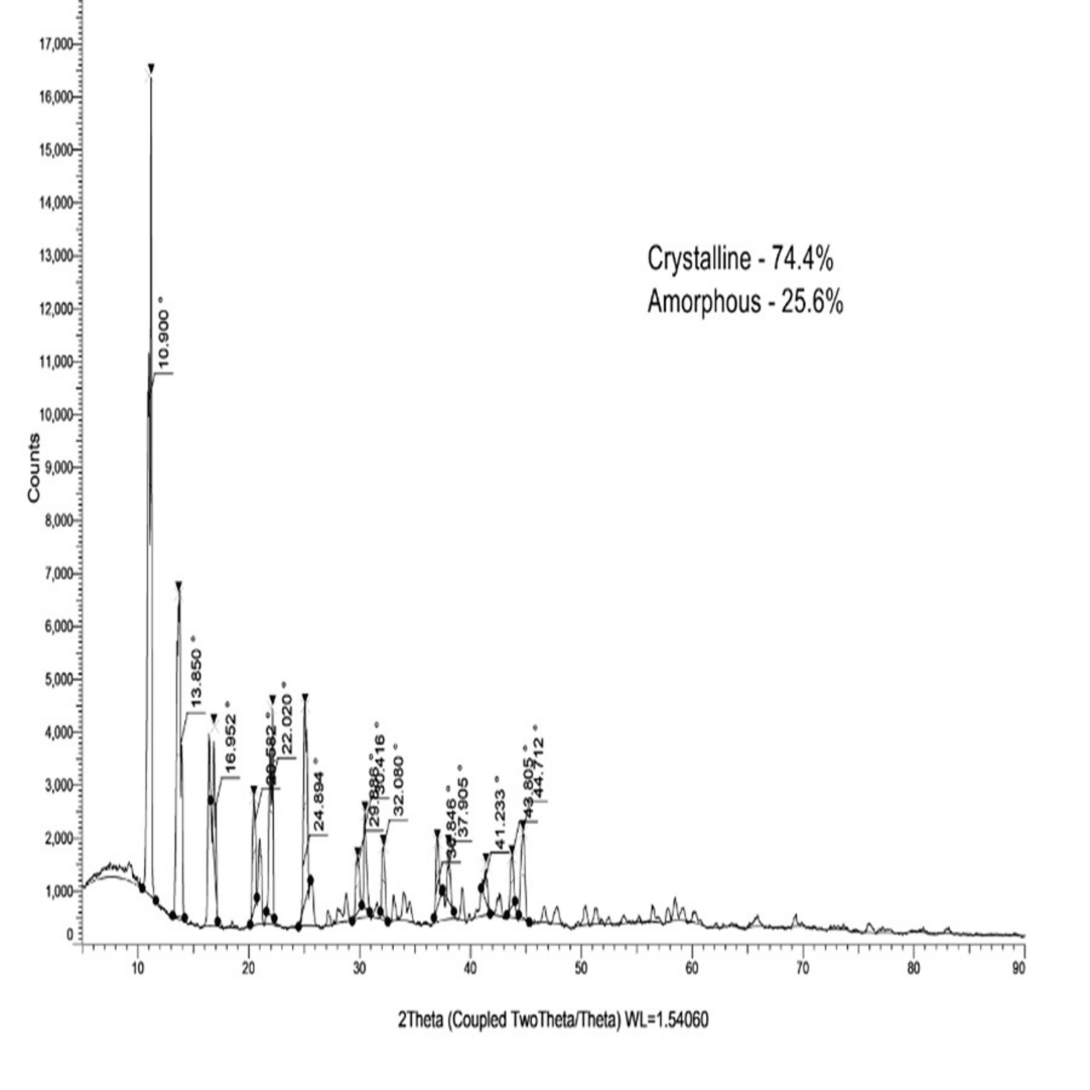
XRD pattern of the as-prepared and annealed (800°C) β-Ch-ZnO-NPs XRD: X-ray diffraction; β-Ch-ZnO-NPs: β-chitosan-derived zinc oxide nanoparticles

Diffraction from the as-prepared and annealed β-Ch-ZnO-NPs samples occurs based on Bragg’s law nλ = 2dsin⁡θ, where n is the integer, λ is the wavelength of Cu Kα1 radiation, d is the interplanar spacing, and θ is the diffraction angle. The output from XRD analysis of the as-prepared and annealed β-Ch-Zn O-NPs samples yields a plot of intensity versus angle of diffraction, as shown in Figure 3. The β-Ch-ZO-NPs exhibit several diffraction peaks, which can be indexed to the crystalline zinc oxide phase with specific lattice parameters. No diffraction peaks corresponding to unreacted zinc, zinc oxides, or other phases were detected, indicating that pure ZnONPs were formed.

Effect of β-Ch-ZnO-NPs on cell viability

**Figure 4:**
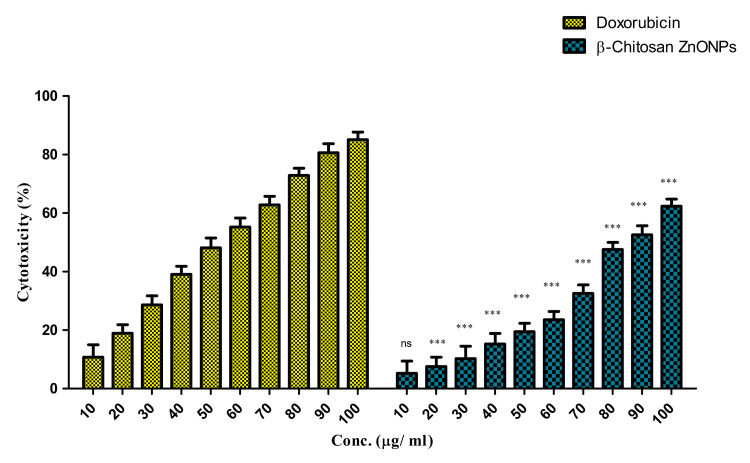
Cytotoxicity of β-Ch-ZnO-NPs on McCoy cells The statistical significance was analyzed with the t-test and the Mann-Whitney U test (compared every concentration of the control group to each concentration of the treatment group). A p value of less than 0.05 was considered statistically significant. ***p < 0.001: extremely statistically significant; ns: nonsignificant β-Ch-ZnO-NPs: β-chitosan-derived zinc oxide nanoparticles Image credit: Meenakshi Sundaram K

The MTT assay evaluated the cytotoxicity of zinc nanoparticles conjugated with β-chitosan on McCoy cells. The cells were treated with varying concentrations of nanoparticles (10, 20, 30, 40, 50, 60, 70, 80, 90, and 100 μg/mL) for 24 hours. Doxorubicin was used as the positive control. The viability of untreated McCoy cells served as a control (100% viability). The results showed that doxorubicin and β-CT-ZnO NPs showed similar cytotoxic patterns on the treated cells. The cytotoxicity was concentration-dependent (Figure 4). The IC_50 _value for the β-CT-ZnO NPs was 330 ± 5 μg/mL. Significant differences were observed at most of the concentrations in the comparison between the doxorubicin and β-CT-ZnO NPs. At the lowest concentration (10 µg/mL), the difference in values was -5.55 with a p value >0.05, indicating no significant difference. However, from concentrations of 20 µg/mL and beyond, none of the β-CT-ZnO NPs consistently showed significantly lower values compared to doxorubicin, with all p values being <0.001. Further, the 95% confidence intervals for this difference were entirely below zero, confirming the statistical significance. These results suggested that β-CT-ZnO NPs exhibited a more pronounced effect than doxorubicin at all significant concentrations, indicating superior efficacy in the measured parameter. Hence, two concentrations, 33 and 66 μg/mL, were used to evaluate the gene expression pattern by RT-PCR method in further experiments. The t-test and Mann- Whitney test showed that both groups (doxorubicin and β-CT-ZnO NPs) showed significant differences at higher concentrations (for both tests, p < 0.05). Hence, 33 and 66 μg/mL were selected for analyzing the gene expression ratio based on the statistical data (data not shown).

To investigate the anti-inflammatory effects of β-CT-ZnO NPs, McCoy cells were stimulated with LPS to induce an inflammatory response, followed by the treatment with β-Ch-Zn NPs. The expression levels of key proinflammatory and anti-inflammatory cytokines were measured using q RT-PCR. The results were normalized to β-actin expression and analyzed using the 2^-ΔΔCt^ method. Both the t-test and Mann-Whitney U test showed significant differences between the control group and the 33 and 66 µg/ml treatment groups for all biomarkers. This indicated that the treatment at both concentrations significantly affects the level of these biomarkers compared to the control group.

IL-2 expression

**Figure 5:**
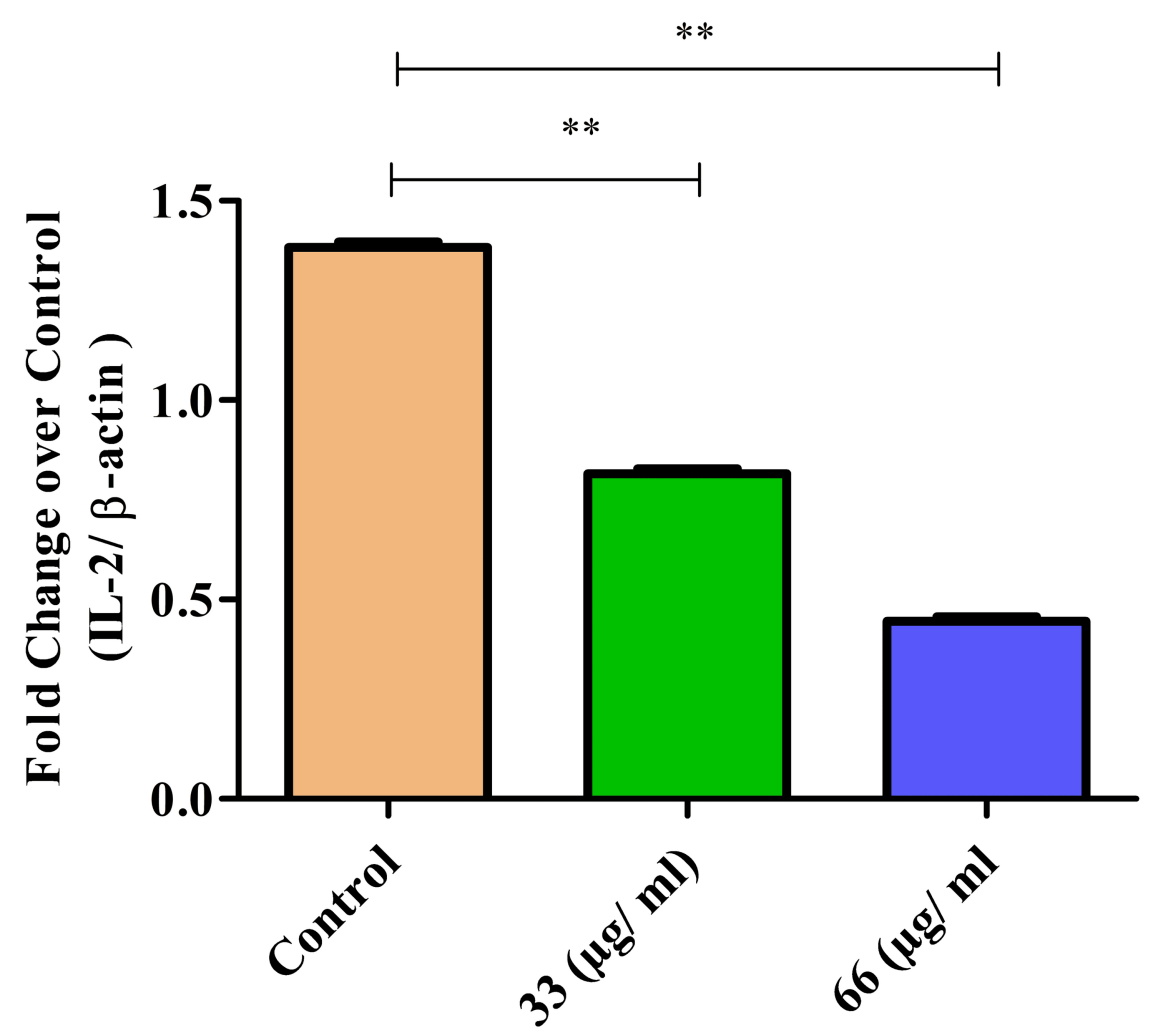
β-Ch-ZnO-NPs decreased IL-2 expression on McCoy cells in a concentration-dependent manner The statistical significance was analyzed with the t-test and the Mann-Whitney U test (comparing the control group to each treatment group). A p value of less than 0.05 was considered statistically significant. **p = 0.0079: statistically significant IL-2: interleukin 2; β-Ch-ZnO-NPs: β-chitosan-derived zinc oxide nanoparticles Image credit: Meenakshi Sundaram K

IL-2 is pivotal in immunotherapy by stimulating T-cell proliferation and activation, enhancing immune responses against cancer cells. Treatment with β-CT-ZnO NPs significantly suppressed IL-2 expression in a dose-dependent manner. The highest concentration of β-Ch-Zn NPs reduced the IL-2 expression by twofold (Figure 5). A significant reduction in IL-2 expression was observed at 33 and 63 µg/mL compared to control groups (p < 0.01 for both comparisons), indicating a suppressive effect. T-test and Mann-Whitney U test revealed that the p value was 0.011 for both groups.

IL-6 expression

**Figure 6:**
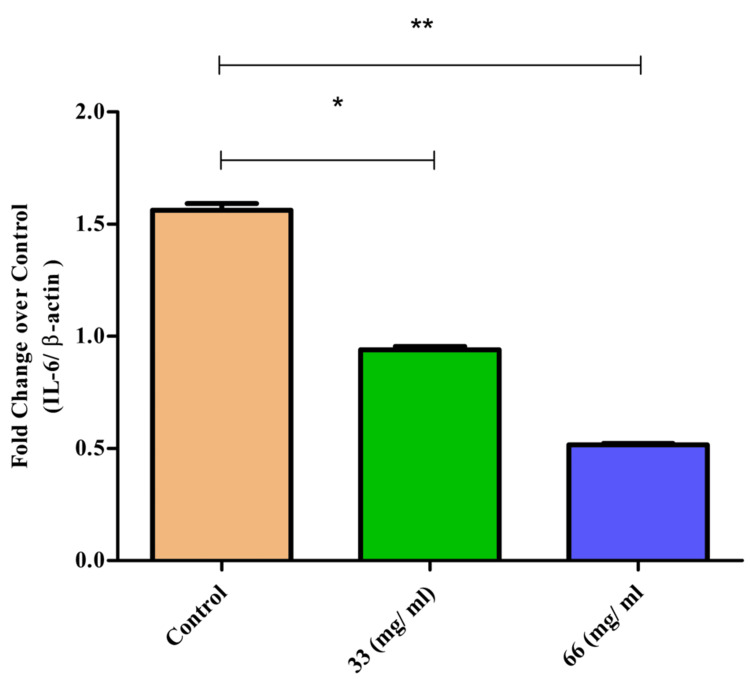
β-Ch-ZnO-NPs decreased IL-6 expression on McCoy cells in a concentration-dependent manner The values were represented as mean ± standard deviation. The statistical significance was analyzed using the t-test and the Mann-Whitney U test. A p value of less than 0.05 was considered statistically significant. *p = 0.05: statistically significant; **p = 0.01: highly statistically significant IL-6: interleukin 6; β-Ch-ZnO-NPs: β-chitosan-derived zinc oxide nanoparticles Image credit: Meenakshi Sundaram K

IL-6 is another critical proinflammatory cytokine whose expression was significantly elevated upon LPS stimulation. Treatment with β-CT-ZnO NPs significantly suppressed IL-6 expression in a dose-dependent manner. The highest concentration of β-CT-ZnO NPs reduced the IL-6 expression by about 45%. This suppression indicates that β-CT-ZnO NPs can modulate the inflammatory environment by inhibiting the production of IL-6, thereby reducing inflammation (Figure 6). Significant downregulation was noted at 33 µg/mL (p = 0.01, denoted by *) for IL6 expression, indicating an effective reduction in inflammatory cytokine production. Meanwhile, the 33 µg/mL treatment showed highly statistically significant downregulation (p = 0.007, denoted by **) on the t-test and Mann-Whitney U test. These results supported the concentration-dependent activity of β-CT-ZnO NPs.

HIF expression

**Figure 7:**
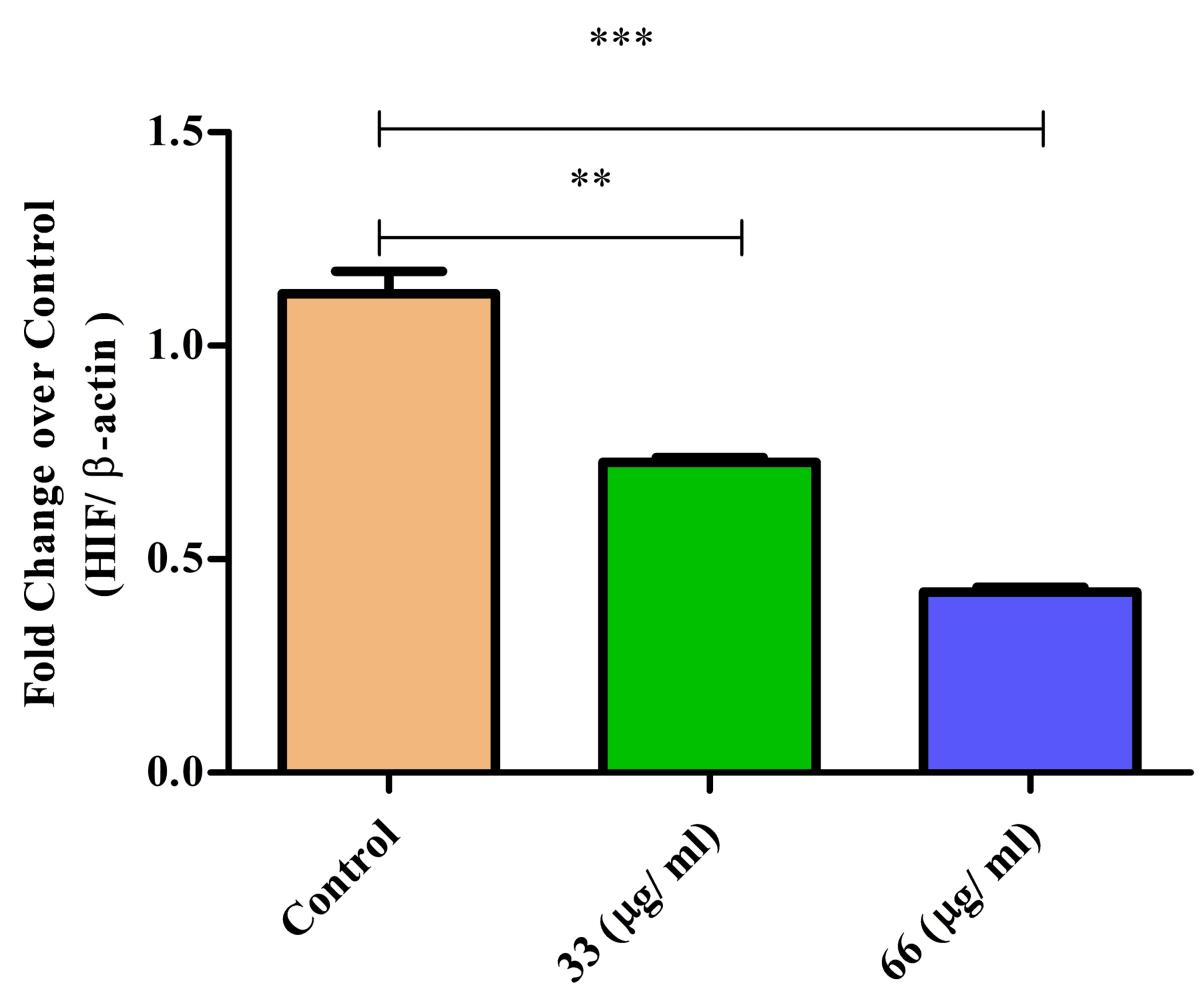
β-Ch-ZnO-NPs decreased HIF expression on McCoy cells in a concentration-dependent manner The values were represented as mean ± standard deviation. The statistical significance was analyzed using the t-test and the Mann-Whitney U test. A p value of less than 0.05 was considered statistically significant. **p = 0.01: highly statistically significant; ***p = 0.008: extremely statistically significant HIF: hypoxia-inducible factor; β-Ch-ZnO-NPs: β-chitosan-derived zinc oxide nanoparticles Image credit: Meenakshi Sundaram K

HIF expression in cancer biology refers to the upregulation of these transcription factors in response to low oxygen levels (hypoxia) within tumors. This adaptive response promotes tumor survival, angiogenesis, metastasis, and resistance to therapy, making HIFs crucial targets for cancer treatment strategies. Treatment with β-CT-ZnO NPs significantly suppressed HIF expression in a dose-dependent manner. The highest concentration of β-Ch-Zn NPs reduced the HIF expression (Figure 7). The expression of HIF level was significantly lower in both 33 and 66 µg/mL groups compared to the control, and the most pronounced reduction was observed at 66 µg/mL (p < 0.01, p = 0.008, t-test and Mann- Whitney U test). This indicated that higher treatment concentrations reduced HIF expression, which might reflect on anti-inflammatory response.

NF-κB expression

**Figure 8:**
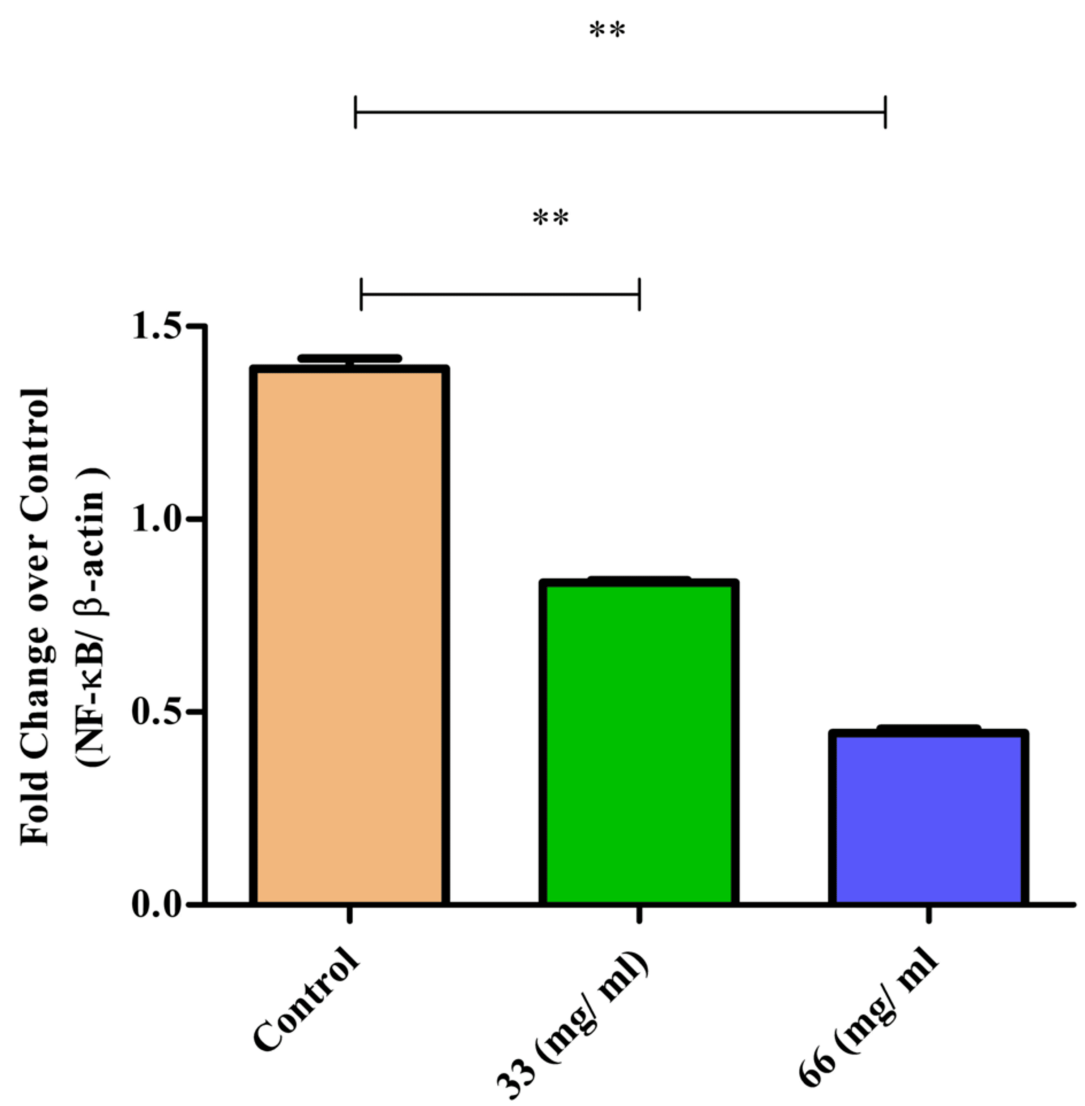
β-Ch-ZnO-NPs decreased NF-κB expression on McCoy cells in a concentration-dependent manner The values were represented as mean ± standard deviation. The statistical significance was analyzed using the t-test and the Mann-Whitney U test. A p value of less than 0.05 was considered statistically significant. **p = 0.01: highly statistically significant NF-κB: nuclear factor kappa B; β-Ch-ZnO-NPs: β-chitosan-derived zinc oxide nanoparticles Image credit: Meenakshi Sundaram K

The NF-κB pathway plays a central role in regulating the expression of various proinflammatory cytokines. LPS stimulation led to a significant increase in NF-κB expression in McCoy cells. However, β-Ch-Zn NPs treatment resulted in a substantial decrease in NF-κB expression. The reduction was most pronounced at 66 µg/mL, where NF-κB levels were lowered by approximately 50%. This suggests that β-CT-ZnO NPs can effectively inhibit the activation of the NF-κB pathway, thereby attenuating the overall inflammatory response (Figure 8). Both 33 and 66 µg/mL treatment resulted in significantly lower NF- κβ expression, with the effect being most pronounced at 66 µg/mL (p < 0.01). This suggested that the treatments effectively suppressed nuclear factor property signaling, which is crucial for inflammatory response regulation.

## Discussion

The modulation of inflammation in McCoy cells using ZnO nanoparticles conjugated with β-chitosan signifies a promising approach for managing inflammatory conditions. LPS-induced inflammation in McCoy cells is a well-established model for studying inflammatory responses mimicking aspects of bacterial infection and inflammation. This discussion explores the potential of β-CT-ZnO NPs in modulating this inflammatory response, drawing on recent literature to provide a comprehensive analysis.

 LPS-induced inflammation in McCoy cells is characterized by the release of proinflammatory cytokines, including TNF-α, IL-1β, and IL-6, as well as oxidative stress and activation of NF-κB signaling pathways [20-26]. β-chitosan polymer derived from chitin has shown anti-inflammatory properties due to its ability to interact with the cellular receptors and modulate inflammatory signaling pathways. Zinc nanoparticles, on the other hand, possess antagonistic properties that can mitigate oxidative stress and inflammation [21,24,26]. The conjugation of β-chitosan with ZnONPs was undertaken to optimize the benefits of both compounds, with the goal of enhancing the stability and bioavailability of ZnONPs. Additionally, this process aimed to capitalize on the size-dependent properties of ZnONPs, thereby improving their ability to penetrate cells more effectively [27]. Recent studies have demonstrated that such conjugation improves the targeting of nanoparticles to inflammatory sites and enhances their therapeutic efficacy [26-28].

 Recent literature indicated that β-chitosan in conjugated zinc nanoparticles can significantly modulate inflammatory responses. A study by β-chitosan coated ZnONPs demonstrated superior anti-inflammatory effects compared to free ZnONPs or β-chitosan alone. The authors attributed this to the enhanced cellular updates and targeted delivery of ZnONPs due to chitosan coatings [24-28]. Similarly, Lee et al. found that β-chitosan with ZnO nanoparticles effectively reduced LPS-induced cytokine production in macrophage cell lines, including McCoy cells, by inhibiting the NF-κB pathway [29]. The inflammatory effects of β-CT-ZnO NPs are mediated through several mechanisms. First, β-chitosan is known to inhibit activation of NF-κB, a key transcription factor involved in inflammation [20-23]. By conjugating zinc with β-chitosan, the nanoparticles can better modulate this pathway, leading to reduced expression of proinflammatory cytokines. Second, ZnNPs have intrinsic antioxidative properties that further help reduce oxidative stress associated with inflammation. The combined effect of β-chitosan and ZnONPs results in a synergistic anti-inflammatory response [26-29]. Moreover, the use of β-chitosan as a carrier for ZnONPs improves their stability and reduces potential cytotoxicity [27]. Recent advancements in nanoparticle formation have emphasized the importance of bioavailability and minimal side effects in therapeutic applications. β-chitosan not only stabilizes zinc or particles but also facilitates their controlled release, ensuring sustained anti-inflammatory effects without harm to healthy cells [29]. The present study showed that β-chitosan in conjugated ZnO had promising activity in the modulation of inflammatory pathways in McCoy cells. Future research should address challenges such as optimizing formulations and conducting comprehensive individual studies to evaluate the full therapeutic potential of the nanoparticle.

## Conclusions

The study demonstrates that biosynthetically derived β-Ch-Zn NPs exhibit significant anti-inflammatory effects on McCoy cells by modulating the expression of key cytokines and signaling pathways. These findings provide a strong foundation for further exploration of β-Ch-Zn NPs as potential therapeutic agents for managing inflammatory diseases. Further studies could focus on in vivo evaluations and the exploration of the underlying molecular mechanisms to fully elucidate the therapeutic potential of these nanoparticles.
